# Ultra-sensitive gas sensor based fano resonance modes in periodic and fibonacci quasi-periodic Pt/PtS_2_ structures

**DOI:** 10.1038/s41598-022-13898-4

**Published:** 2022-06-13

**Authors:** Shrouk E. Zaki, Mohamed A. Basyooni

**Affiliations:** 1 Department of Nanotechnology and Advanced Materials, Graduate School of Applied and Natural Science, Selçuk University, Konya, 42030 Turkey; 2 Science and Technology Research and Application Center (BITAM), Necmettin Erbakan University, Konya, 42090 Turkey; 3 Theoretical Physics Department, National Research Center, Dokki, Cairo Egypt

**Keywords:** Computational methods, Computational science

## Abstract

Ultra-sensitive greenhouse gas sensors for CO_2_, N_2_O, and CH_4_ gases based on Fano resonance modes have been observed through periodic and quasi-periodic phononic crystal structures. We introduced a novel composite based on metal/2D transition metal dichalcogenides (TMDs), namely; platinum/platinum disulfide (Pt/PtS_2_) composite materials. Our gas sensors were built based on the periodic and quasi-periodic phononic crystal structures of simple Fibonacci (F(5)) and generalized Fibonacci (FC(7, 1)) quasi-periodic phononic crystal structures. The FC(7, 1) structure represented the highest sensitivity for CO_2_, N_2_O, and CH_4_ gases compared to periodic and F(5) phononic crystal structures. Moreover, very sharp Fano resonance modes were observed for the first time in the investigated gas sensor structures, resulting in high Fano resonance frequency, novel sensitivity, quality factor, and figure of merit values for all gases. The FC(7, 1) quasi-periodic structure introduced the best layer sequences for ultra-sensitive phononic crystal greenhouse gas sensors. The highest sensitivity was introduced by FC(7, 1) quasiperiodic structure for the CH_4_ with a value of 2.059 (GHz/m.s^−1^). Further, the temperature effect on the position of Fano resonance modes introduced by FC(7, 1) quasi-periodic PhC gas sensor towards CH_4_ gas has been introduced in detail. The results show the highest sensitivity at 70 °C with a value of 13.3 (GHz/°C). Moreover, the highest Q and FOM recorded towards CH_4_ have values of 7809 and 78.1 (m.s^−1^)^−1^ respectively at 100 °C.

## Introduction

Nowadays, the trace of gas sensing especially toxic and greenhouse gases has great attention for a wide variety of practical applications, such as medical inspection, environmental monitoring, and production control^1^. For various toxic gases, a variety of physical and chemical sensing systems have been devised^2^. For example, a variety of sensors for detecting carbon dioxide (CO_2_) have been created, including a phononic crystal (PhC)^2^, catalytic^3^, fluorescent^4^, and semiconductor thin films^5–11^. Greenhouses gases such as CO_2_, N_2_O, and CH_4_ are regarded as extremely dangerous gases because they absorb infrared radiation (IR) emitted from the Earth’s surface and reradiate it back to the Earth’s surface, trapping heat in the Earth’s atmosphere^11,12^. Gas sensors employ a variety of operating principles based on various methods, including catalytic^3^, semiconductor thin films^5–11^, and optical gas sensors^13^. Another approach is to use acoustic waves in gas sensing^2^ because the sound speed of a binary gas mixture varies depending on its composition^14,15^. As a result, the phononic composite structures were introduced as acoustic gas sensors promising for experimental and low-cost with no lead time sensing application^2^. PhCs are introduced as unique artificial structures built of a periodic replication of scatterers in a matrix that allows controlling and modifying the input mechanical and acoustical waves^16^. The PhC based systems are considered an ideal candidate for developing acoustic gas sensors. Kushwaha first introduced the PhCs concept with the ability to manipulate mechanical waves to trap, transmit, or prevent their propagation at specific frequency ranges^17^. Moreover, the novel property of PhC is the phononic bandgap (PhC-BG). It can be formed when the reflected waves interfere constructively at the interface between alternating periodic layers and so-called stop PhC band gaps. Meanwhile, when the acoustic waves propagate through the PhC freely the destructive interference occurs and the passband gaps appeared^18^. Mechanical filters, noise suppression, sensors, ultrasonic imaging devices, and acoustic diodes are just a few of the applications that can be derived from PhC-BG properties^18–22^. However, one of the benefits of using PhC structures is the ability to modulate any external influence such as pressure or temperature, on the reference and target gases^23^. Meanwhile, the PhC structure with a cavity filled with different gases and embedded inside has significant advantages over the regular PhCs^24^. For instance, by inserting a cavity inside the PhC the structure periodicity has been broken and several resonance modes will be introduced through PhC-BG, which in turn raised the novelty of such PhC structure more than the regular ones^25^. In the fields of phononics, quasi-periodic structures have recently piqued the interest of researchers^26,27^. The periodic structures with special ordering patterns provide an extra degree of freedom in the design and control of the structure’s characteristics. Quasi-periodic structures have to lack translational symmetry and refer to aperiodic structures with special ordering patterns that introduce an extra degree of freedom in design and control of the structure’s characteristics. Fibonacci, Cantor, Dodecanacci, Rudin Shapiro, and others are some of the different rules that could be used to generate quasi-periodic sequences^26,28^. In this way, quasi-periodic structures may be more effective than periodic designs in constructing omnidirectional band gaps and providing wide phononic and photonic band gaps. In addition, they are the best choice for tuning transmission modes and are capable of easily creating waveguides and cavities in PnCs^29^. So far, the quasi-periodic structures in 1D and 2D PhCs have been achieved for both solid–solid and solid–fluid structures^28,30^. In quasi-periodic structures, band gaps with strong resonances peaks are also expected, which can strongly localize acoustic waves^31^. As a result, the quasi-periodic PhCs are promising candidates for overcoming the low-frequency limitations of large-scale acoustic structures^30^.

Artificial and natural resonators full our life ranging from lasers to complicated systems including the musical devices and machines of imaging^32^. Moreover, recently the appearance of the Fano resonance mode inside the PhC-BG of the PhC sensors structures has significant attention due to their line shape being asymmetric and sharp^33^. Fano resonances have attracted a lot of attention since they first appeared more than fifty years ago, owing to their sharp asymmetric line shape, which is caused by destructive interference between narrow discrete states and broad continuum states^34,35^. It attracts great attention because of its wide range of applications in sensors and optical devices^36^. In PhC periodic structures, the Fano resonance phenomenon has been studied; also it has been used in PhC structure acoustic waveguide techniques^37^. The Fano resonance used in several phononics applications includes PhC resonators^38^, waveguiding^39^, and radiation detectors^40^. Meanwhile, the Fano resonance-based periodic and quasi-periodic PhC gas sensor structures, in which a very shark resonance transmitted modes with novel sensitivity, quality factors, and figure of merit did not cover before. Furthermore, the Fano resonance phenomena don’t introduce in all previous literature of 1D or 2D PhCs gas sensors^2,14,41–43^. According to the previous literature, Cicek et al. proposed experimentally a PhC structure to use as an acoustic gas sensor^2^. In addition, they introduced an acoustic gas sensor by using PhC for CO_2_^41^. Also, Cheeke et al. introduced the acoustic wave with gases interaction^14^. In addition, Shrouk et al. designed defected PhC as a gas sensor theoretically to detect toxic gases including CH_4_, O_2_, CO_2_, and NH_3_^16^. Further, Hadiseh et al. demonstrated periodic and quasi-periodic structures as a gas sensor towards NH_3_, CH_4_, O_2_, and CO_2_^31^. Furthermore, Kaya et al. proposed experimentally and numerically a 1D-PhC gas sensor to obtain CO_2_ levels in the air^42^.

In this work, firstly, we proposed a study that covered the sensing of several greenhouse gases including CO_2_, N_2_O, and CH_4_ based on periodic and quasi-periodic PhC structures. Secondly, it is the first time to show a very sharp Fano resonance mode within the PhC-BG of the gas sensors structures. These Fano resonance modes introduced by periodic and quasi-periodic PhC structures achieved novel sensitivity, figure-of-merit, and quality factor values towards target greenhouse gases. Thirdly, the temperature effect on the position of Fano resonance modes introduced by FC(7, 1) quasi-periodic PhC gas sensor towards CH_4_ gas has been introduced in detail. Moreover, we studied the effect of temperature on the sensitivity, quality factor, and figure of merit of FC(7, 1) quasi-periodic PhC gas sensor.

## Materials and mechanism

### PhC gas sensor structures and mechanism

**Figure 1:**
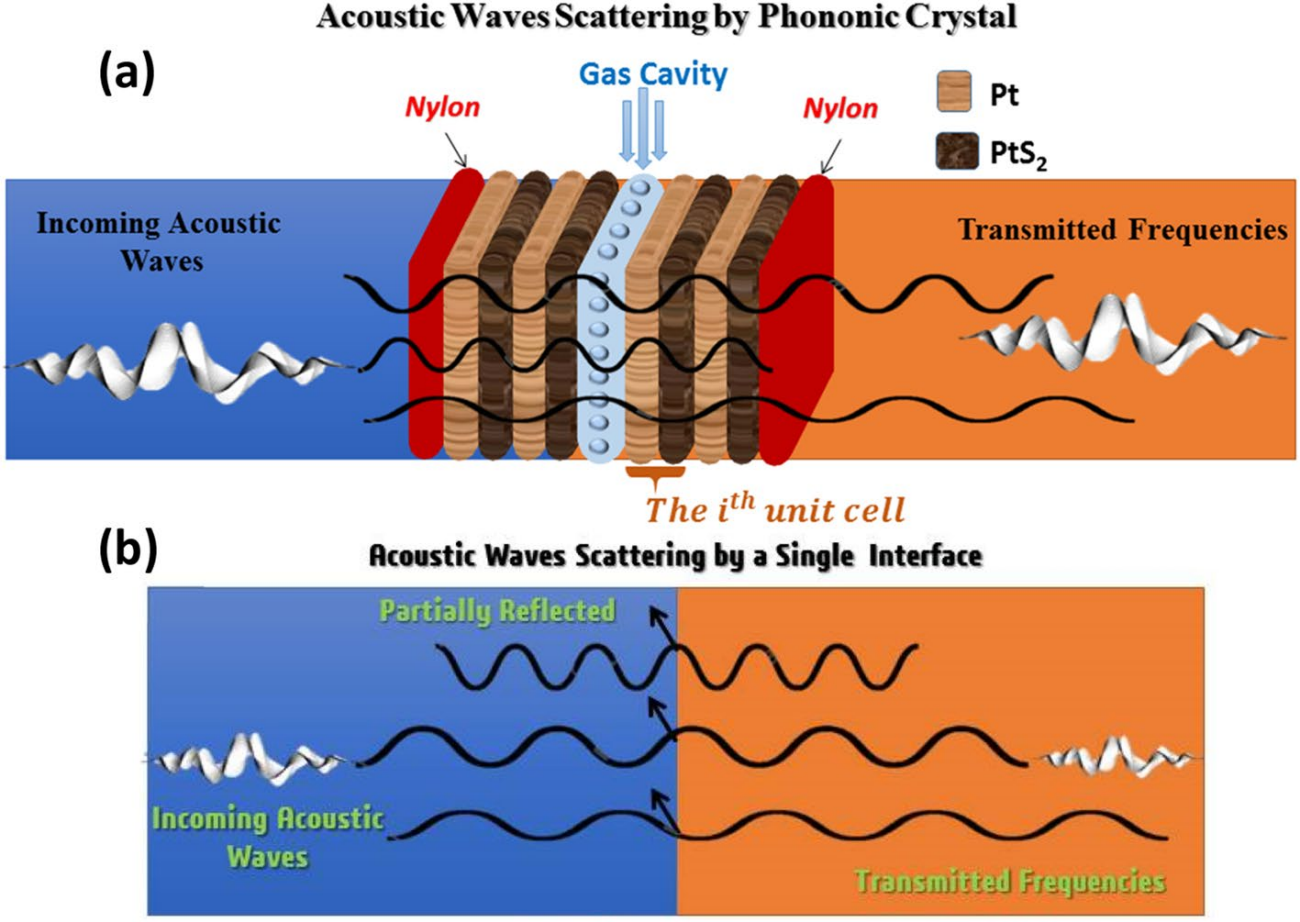
(**a**) The Mechanism of interaction acoustic waves through PhC greenhouse gas sensor structures, (**b**) the attenuation of the incident acoustic waves within the interface between two layers of the structures.

**Figure 2:**
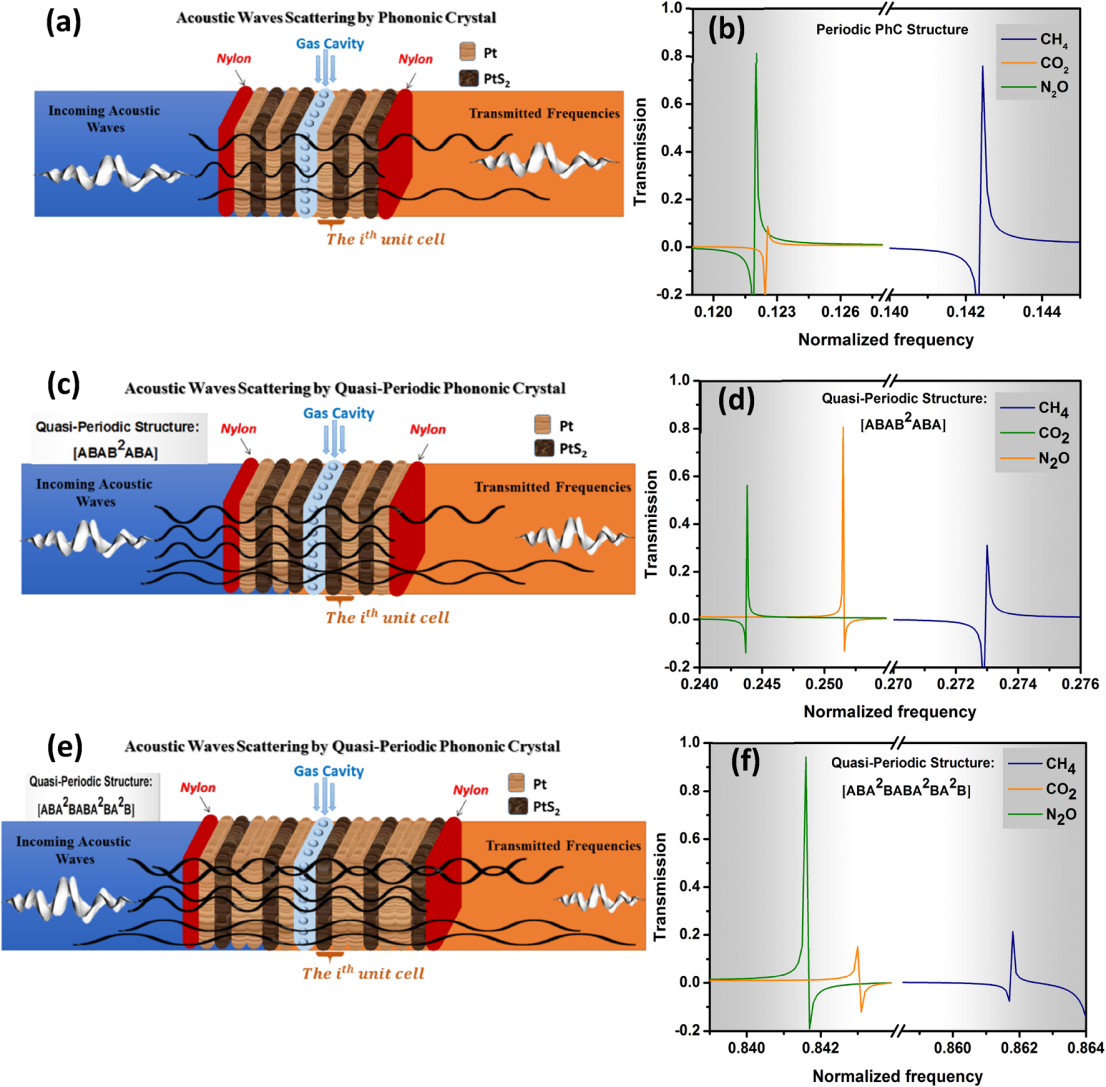
(**a**, **c**, **e**) introduced the periodic and F(5), FC(7, 1) quasi-periodic PhC gas sensor structures respectively, (**b**, **d**, **f**) Fano resonance transmitted spectra versus normalized frequency towards N_2_O, CH_4_, and CO_2_ greenhouse gases at room temperature of periodic and F(5), FC(7, 1) quasi-periodic PhC gas sensor structures respectively.

We introduced the interaction between the acoustic sound waves and the PhC multilayers structure, as observed in Fig. 1a, b. In this work, we study periodic PhC structure and two quasi-periodic PhC as a greenhouse gas sensor towards N_2_O, CH_4_, and CO_2_ gases for the first time. In the case of the periodic PhC structure it composites of altogether 8 layers immersed between two layers of Nylon as [(Nylon)(A/B)^2^−(greenhouse gas)−(A/B)^2^)(Nylon)], where A/B is a repetition of two solid layers of Pt /PtS_2_. The second structure is F(5) quasi-periodic PhC structure with layers sequence of [ABAB^2^ABA], and the third one is FC(7, 1) quasi-periodic PhC structure with layers sequence of [ABA^2^BABA^2^BA^2^B] as shown in Table 1. The periodic and F(5) quasi-periodic structures have the same layers number, and the same thickness d_(periodic)_ = d_(F(5))_ = d_(FC(7,1))_ = 1 nm. Meanwhile, the FC(7, 1) quasi-periodic structure has a large number of layers than others. In the middle of these structures, we inserted a cavity that will be filled with target greenhouse gases separately. Table 2 shows the acoustic properties of the structure materials. We utilized Pt and PtS_2_ because of the enormous impedance mismatch between them. The acoustic properties of the developed layers and the gas-filled cavity are the keys to be taken as input parameters and demonstrate its gas sensor attempt. The sound speed and mass density are the acoustic properties that express these properties. Our mechanism introduced the ability of periodic and quasi-periodic PhC structures to detect greenhouse gases with significant sensitivity for each gas. For the periodic PhC structure, the acoustic properties of the structural layers such as acoustic sound speed and density are changing regularly, due to the periodicity of PhC as demonstrated in Fig. 1a. As a result, the incident acoustic waves are scattered within the structure at the interface between every two layers as observed in Fig. 1b. If the interference was constructive, it causes the establishment of the blocked PhC band gaps^44,45^. Meanwhile, if the interference is destructive, the resultant band is a transmission band^24,46^. The wave equation of the incident acoustic wave was given in Eq. (1) in the theoretical treatment part. Also, Eq. (11) shows the coefficient of transmitted waves that introduces the transmission of acoustic waves within the PhC structures. On the other side, the F(5) and FC(7, 1) quasi-periodic PhC structures with layers sequence of [ABAB^2^ABA], and [ABA^2^BABA^2^BA^2^B] respectively, have a lack translational symmetry and introduce periodic structures with special ordering patterns, which in turns leads to more and more attenuation for the propagation of the acoustic wave through the structures due to extra degree of freedom for the propagation of acoustic waves within^28,30^. For the interaction between acoustic waves and greenhouse gases that filled separately in a cavity inside PhC, the cavity can confine some energy of the incident acoustic wave introduced in the generation of Fano resonance peaks inside the PhC-BG related to each gas as illustrated in Fig. 2b, d, f. By changing the greenhouse gas type, the intensity and frequency of the Fano resonance peak will be altered as well. These Fano resonance peaks introduce the ability of our three PhC structures to sense the greenhouse gases efficiently. Moreover, it can demonstrate the physical properties and type of the target greenhouse gases with significant sensitivity, quality factor, and figure of merit.

**Table 1:** The layers sequences of the quasi-periodic PhCs structures^31^.

Structure	Layer sequence
F(5)	ABAB^2^ABA
FC(7,1)	ABA^2^BABA^2^BA^2^B

**Table 2:** Shows the acoustic properties values of the materials of the structure used in this work^16,31^.

Materials	Density (kg/m^3^)	Acoustic sound speed (m/s)	Thickness
PtS_2_	10,760	1960	1 nm
Pt	1140	2770	1 nm
Sensing Greenhouse Gases			
CO_2_	1.8393	267	1.5 nm
N_2_O	0.7069	430	1.5 nm
CH_4_	0.659	445	1.5 nm
Air (Reference)	1.2047	343	1.5 nm

### Theoretical treatment

The binary structures PhC gas sensor was proposed in this work as introduced in Fig. 2a, c, e. Recently, the periodic and quasi-periodic PhC structures attracted great attention, because they can introduce a high performance for sensing applications compared to the regular PhC structures^26,27,47,48^. The transfer matrix method (TMM) is used to provide the transmission and reflection of acoustic sound waves within the multilayer PhC systems^49^. The ${d}_{j}$ is the thickness of layer ${x}_{j}.$ at the interfaces between structure layers the continuity conditions of acoustic wave propagation were taken into account. The acoustic properties of the layers, such as acoustic speed and mass density, change periodically when an acoustic wave strikes our proposed multilayer PhC structure, and the incident acoustic wave disperses into several waves inside the structure. The Acoustic wave propagation within our multilayer gas sensor PhC structures as shown in Fig. 2a, c, e is introduced by a differential equation as^49,50^.

1 $$ \frac{1}{{C_{j}^{2} }}\frac{{\partial^{2} p}}{{\partial t^{2} }} - \nabla^{2} p = 0 $$

where $C_{i } {\text{is}}$ the acoustic sound speed within the layer j, the subscript j = 1, and 2, shows the layer type and $p$ is the acoustic wave pressure. The solution of Eq. (1) will be shown by Eq. (2):

2 $$ p_{j} = \left( {A_{ + }^{\left( j \right)} e^{{ + iK_{j} X}} + A_{ - }^{\left( j \right)} e^{{ - iK_{j} X}} } \right)e^{i\omega t} $$

where the $A_{ + }^{\left( j \right)} {\text{and}} A_{ - }^{\left( j \right)}$ are the transmitted and reflected waves amplitudes respectively, $\omega$ is the angular frequency of the propagation waves and $K_{j} = \omega /C_{j}$ is wave vector which depends on the acoustic sound speed of waves through the structure layers.

According to the interaction of acoustic waves with our structures, at the interface between every two layers, the acoustic stress and displacement continuity should be obtained. The stress caused by acoustic waves through our structures can be demonstrated the Eq. (3)^16,51,52^:

3 $$ \sigma = E_{j} \frac{{\partial p_{j} }}{{\partial {\text{x}}}} $$

where $E_{j}$ is Young’s modulus of each material built the PhC structure. By substituting Eq. (2) into Eq. (3) we can show the stress as:

4 $$ \begin{gathered} \sigma \left( x \right) = iE_{j} K_{j} \left[ {A_{ + }^{\left( j \right)} e^{{ + iK_{j} X}} - A_{ - }^{\left( j \right)} e^{{ - iK_{j} X}} } \right] \hfill \\ \sigma \left( x \right) = iZ_{j} \left[ {A_{ + }^{\left( j \right)} e^{{ + iK_{j} X}} - A_{ - }^{\left( j \right)} e^{{ - iK_{j} X}} } \right] \hfill \\ \end{gathered} $$

where $Z_{j} = E_{j} K_{j}$ indicates the acoustic impedance. We can rewrite the Eq. (4) components as given in Eq. (5):

5 $$ \left[ {\begin{array}{*{20}c} {{\text{u}}\left( {\text{x}} \right)} \\ {\sigma \left( {\text{x}} \right)} \\ \end{array} } \right] = \left[ {\begin{array}{*{20}c} 1 & 1 \\ {iZ_{j} } & {iZ_{j} } \\ \end{array} } \right]\left[ {\begin{array}{*{20}c} {A_{ + }^{\left( j \right)} e^{{ + iK_{j} X}} } \\ {A_{ - }^{\left( j \right)} e^{{ - iK_{j} X}} } \\ \end{array} } \right] = \left[ {\begin{array}{*{20}c} {A_{ + }^{\left( j \right)} e^{{ + iK_{j} X}} } \\ {A_{ - }^{\left( j \right)} e^{{ - iK_{j} X}} } \\ \end{array} } \right] $$

where $$B_{j}$$ is the wave matrix at the interface between two layers. These components enable us to use the relation of $$X_{R}^{j}$$ = $$X_{L}^{j} + d_{j}$$ where $$X_{R}^{j}$$ and $$X_{L}^{j}$$ represented the right and left boundary position, respectively, of each layer (*j*). As a result, the displacement and stress are related from $$X_{L}^{j}$$ to those at $$ X_{R}^{j}$$ as seen in Eq. (6):

6 $$ \begin{gathered}   \left[ {\begin{array}{*{20}c}    {{\text{u}}\left( {X_{R}^{j} } \right)}  \\    {\sigma \left( {X_{R}^{j} } \right)}  \\   \end{array} } \right] = \left[ {\begin{array}{*{20}c}    {e^{{ + iK_{j} d_{j} }} } & 0  \\    0 & {e^{{ - iK_{j} d_{j} }} }  \\   \end{array} } \right]B_{j} \left[ {\begin{array}{*{20}c}    {A_{ + }^{{\left( j \right)}} e^{{ + iK_{j} X_{L}^{j} {\text{~}}}} }  \\    {A_{ - }^{{\left( j \right)}} e^{{ - iK_{j} X_{L}^{j} {\text{~}}}} }  \\   \end{array} } \right] = ~P_{j} ~B_{j} \left[ {\begin{array}{*{20}c}    {A_{ + }^{{\left( j \right)}} e^{{ + iK_{j} X_{L}^{j} {\text{~}}}} }  \\    {A_{ - }^{{\left( j \right)}} e^{{ - iK_{j} X_{L}^{j} {\text{~}}}} }  \\   \end{array} } \right] \hfill \\   {\text{And}},\,\,\left[ {\begin{array}{*{20}c}    {{\text{u}}\left( {X_{L}^{j} } \right)}  \\    {\sigma \left( {X_{L}^{j} } \right)}  \\   \end{array} } \right] = B_{j} \left[ {\begin{array}{*{20}c}    {A_{ + }^{{\left( j \right)}} e^{{ + iK_{j} X_{L}^{j} {\text{~}}}} }  \\    {A_{ - }^{{\left( j \right)}} e^{{ - iK_{j} X_{L}^{j} {\text{~}}}} }  \\   \end{array} } \right] \hfill \\  \end{gathered}  $$

where $$P_{j}$$ = $$\left[ {\begin{array}{*{20}c} {e^{{ + iK_{j} d_{j} }} } & 0 \\ 0 & {e^{{ - iK_{j} d_{j} }} } \\ \end{array} } \right]$$ is propagation matrix through each layer (*j)* that describes the propagation of the acoustic waves through a single layer j with a thickness $$ d_{j }$$ of our multilayer PhC gas structures are obtained as in Eq. (7)^49^:

7 $$ P_{j} = \left[ {\begin{array}{*{20}c} {e^{{ + iK_{j} d_{j} }} } & 0 \\ 0 & {e^{{ - iK_{j} d_{j} }} } \\ \end{array} } \right] $$

From Eqs. (6, 7) can be rewritten as follows:

8 $$ \left[ {\begin{array}{*{20}c} {{\text{u}}\left( {X_{R}^{j} } \right)} \\ {\sigma \left( {X_{R}^{j} } \right)} \\ \end{array} } \right] = P_{j} B_{j} \left[ {\begin{array}{*{20}c} {A_{ + }^{\left( j \right)} e^{{iK_{j} X_{L}^{j} { }}} } \\ {A_{ - }^{\left( j \right)} e^{{ - iK_{j} X_{L}^{j} { }}} } \\ \end{array} } \right] = B_{j} P_{j} B_{j}^{1} \left[ {\begin{array}{*{20}c} {{\text{u}}\left( {X_{L}^{j} } \right)} \\ {\sigma \left( {X_{L}^{j} } \right)} \\ \end{array} } \right] = D_{j} \left[ {\begin{array}{*{20}c} {{\text{u}}\left( {X_{L}^{j} } \right)} \\ {\sigma \left( {X_{L}^{j} } \right)} \\ \end{array} } \right] $$

For the same layer *j*, Eq. (8) relates the stress and displacement at left $$\left( {X_{L}^{j} } \right)$$ to right $$\left( {X_{R}^{j} } \right)$$ and $$ D_{j}$$ is the transfer matrix for layer j, has the form as^52^:

9 $$ D_{j} = \left[ {\begin{array}{*{20}c} {\cos \left( {K_{j} d_{j} } \right)} & {1/Z_{j} \sin \left( {K_{j} d_{j} } \right)} \\ { - Z_{j} \sin \left( {K_{j} d_{j} } \right)} & {\cos \left( {K_{j} d_{j} } \right)} \\ \end{array} } \right] $$

Because the transfer matrix applies to any layer and $X_{L}^{j} \equiv X_{R}^{{\left( {j - 1} \right)}}$, we extend Eq. (9) across multiple structures layers as shown in Eq. (10):

10 $$ \begin{gathered} {\text{y}}\left( {X_{R}^{1} } \right) = D_{1} \,{\text{y}}\left( {X_{L}^{1} } \right) = {\text{y}}\left( {X_{L}^{2} } \right){,}\;{\text{y}}\left( {X_{R}^{2} } \right) = D_{2} \,{\text{y}}\left( {X_{L}^{2} } \right) = D_{2} D_{1} \,{\text{y}}\left( {X_{L}^{1} } \right) = {\text{y}}\left( {X_{L}^{3} } \right), \hfill \\ {\text{y}}\left( {X_{R}^{3} } \right) = D_{3 } \,{\text{y}}\left( {X_{L}^{3} } \right) = D_{3} D_{1} D_{2} \,{\text{y}}\left( {X_{L}^{1} } \right) = {\text{y}}\left( {X_{L}^{4} } \right) \hfill \\ {\text{y}}\left( {X_{R}^{n} } \right) = D_{n} D_{n1} \cdots D_{1} \,{\text{y}}\left( {X_{L}^{1} } \right) = D\,{\text{y}}\left( {X_{L}^{1} } \right) \hfill \\ \end{gathered} $$

Finally, the transfer matrix (D) links the displacement and stress at the left end $$\left( {X = X_{L}^{j} } \right)$$ of the first layer in a unit cell to those at the Nth layer right boundary $$\left( {X = X_{R}^{j} } \right)$$. The $$D_{j} $$ matrix depends on the acoustic impedance $$ Z_{j}$$ and $$E_{j}$$ of each material built the PhC structure. The total transfer matrix components $$D_{ij} = D\left( {i,j} \right) $$ introduced in Eq. (9) can be written as Eqs. (11–13):

11 $$ D_{j} \left( {1,1} \right) = { }D_{j} \left( {2,2} \right) = \cos \left( {K_{j} d_{j} } \right) $$

12 $$ D_{j} \left( {1,2} \right) = 1/Z_{j} \sin \left( {K_{j} d_{j} } \right) $$

13 $$ D_{j} \left( {2,1} \right) = - Z_{j} \sin \left( {K_{j} d_{j} } \right) $$

where $Z_{j} = E_{j} K_{j}$ indicates the acoustic impedance, and $d_{j}$ is the layer thickness. We calculated the transmission coefficient of our PhC structures by using Eq. (14)^53^:

14 $$ \frac{{U_{e} }}{{U_{0} }} = \frac{{2E_{0} \left( {D_{11} D_{22} - D_{12} D_{21} } \right)}}{{E_{0} \left( {D_{11} - E_{e} D_{21} } \right) - \left( {D_{12} - E_{e} D_{22} } \right)}} $$

where $${U}_{0}$$, $${U}_{e}$$ are the amplitudes of the incident and transmitted wave, respectively, and $$ E_{0}$$ and $$E_{e}$$ are the two semi-infinite solids Young’s modulus at the left and right of the PhC structure.

### Analyzed structures

This study introduced the acoustic wave propagation through binary periodic and F(5), FC(7, 1) quasi-periodic structures^31^. The periodic and F(5) quasi-periodic structures have the same layers number, and the same thickness d_(periodic)_ = d_(F(5))_ = d_(FC(7,1))_ = 1 nm. Meanwhile, the FC(7, 1) quasi-periodic structure has a large number of layers than others. The quasi-periodic PhCs structures that we used in this work can be introduced by layer sequences demonstrated in Eqs. (6, 7)^31,54^:

- Simple Fibonacci, F(j):
  15 $$ \begin{gathered} F_{j} = B \hfill \\ F_{1} = A \hfill \\ F_{j + 1} = F_{j - 1} F_{j} , j \ge 1 \hfill \\ \end{gathered} $$
- Generalized Fibonacci, FC(j, n):
  16 $$ \begin{gathered} FC_{1} = B \hfill \\ FC_{2} = B^{n - 1} A \hfill \\ FC_{j} = FC_{j - 1}^{n} FC_{j - 2} , j \ge 3 \hfill \\ \end{gathered} $$

## Results and discussion

### The designs and spectral response of periodic and quasi-periodic phononic crystal structures towards high sensitivity gas sensor

As demonstrated in Fig. 2a–f, we introduced the periodic and quasi-periodic PhC structures as a greenhouse gas sensor. For each structure as shown in Fig. 2a, c, d we calculated the transmission spectrum vs the normalized frequency at room temperature towards N_2_O, CH_4_, and CO_2_ gases and shows the best gas sensor structure between them. The periodic PhC gas sensor is composites of altogether 8 layers immersed between two layers of Nylon as [(Nylon)(A/B)^2^−(greenhouse gas)−(A/B)^2^)(Nylon)] as shown in Fig. 2a. The second structure is quasi-periodic PhC can be seen in Fig. 2c, it has the same number of layers of periodic PhC gas sensor with layers sequence of [ABAB^2^ABA]. On the other side, the third one is a quasi-periodic PhC gas sensor with a layers sequence of [ABA^2^BABA^2^BA^2^B]. A/B is a repetition of two solid layers of Pt and PtS_2_. The periodic and F(5) quasi-periodic structures have the same layers number, and the same thickness d_(periodic)_ = d_(F(5))_ = d_(FC(7,1))_ = 1 nm. Meanwhile, the FC(7, 1) quasi-periodic structure has a large number of layers than others. In our work, we used Pt/PtS_2_ layers with nano thickness as the mismatching in the acoustic impedance between 2D materials layers grew as the thicknesses of their construction layers were reduced, which in turn led to forming a wide range band gaps^55,56^. As a result, when the Pt/PtS_2_ structures layers thickness decreased, it allows appearing of wide phononic band gab at a very high frequency^57^. Thus, using nanolayer thickness caused a strong attenuation for the incident acoustic waves through these layers^58^. To the best of our knowledge, an efficiently sensitive greenhouse gas sensor based on the Fano resonance of a PhC structure has been introduced for N_2_O, CH_4_, and CO_2_ gases for the first time. Essentially, our innovation focuses on introducing a smart greenhouse gas sensor based on the PhC structures that address several aspects that have yet to be addressed in earlier research on 1D or 2D PhC gas sensors^2,14,41–43^. The gas detecting idea is experimentally introduced by using 2D PhC Resonators^2^. In addition, the mechanism of the interaction of acoustic waves and gases is demonstrated here^14^. Further, the hole inside a wall’s mechanical properties is experimentally stated before^23,59^.

However, studying the PhC structures as a greenhouse gas sensor based on Fano-resonance, which is the main emphasis of this study, has yet to be covered. Firstly, to compare between the periodic and quasi-periodic PhC structures we used the same number of layers for the two structures as observed in Fig. 2a, c. As we mentioned in the mechanism part, when the acoustic waves interact with the gas inside the cavity, it can confine some energy of the incident acoustic wave introduced in the generation of Fano resonance peaks related to the gas inside the cavity appeared in the transmitted band gaps as illustrated in Fig. 2b, d, f. The appearance of the Fano resonance peaks shows the ability of our PhC structure to sense the greenhouse gases including N_2_O, CH_4_, and CO_2_ gases. Each Fano resonance peak is related to the acoustic properties of each gas. By changing the greenhouse gas type, the intensity and frequency of the Fano resonance peak will be altered as well as shown in Fig. 2b, d, f. From the Fig. 2b, d, we can see that in the case of F(5) quasi-periodic PhC with layers sequence of [ABAB^2^ABA] gas sensor, the Fano resonance peaks related to N_2_O, CH_4_, and CO_2_ gases are shifted to high frequency compared to the periodic PhC structure. Moreover, with increasing the number of layers in the case of FC(7, 1) quasi-periodic structure with layers sequence of [ABA^2^BABA^2^BA^2^B] as seen in Fig. 2e we can see that the Fano resonance peaks of N_2_O, CH_4_, and CO_2_ gases are shifted to more high frequency than the other two structures as introduced Fig. 2b, d. Thus, due to the FC(7, 1), quasi-periodic structures with large layer numbers have a lack of translational symmetry that introduces an extra degree of freedom in design and control of the structure’s characteristics. Based on this result, the FC(7, 1) quasi-periodic structure was introduced as the best greenhouse gas sensor compared to the periodic PhC and F(5) quasi-periodic PhC. Between the N_2_O, CH_4_, and CO_2_ gases, the CH_4_ has been recorded as the highest normalized frequency followed by CO_2_, and N_2_O for the periodic and quasi-periodic PhC structures. In the case of periodic PhC structure, the CH_4_ has a Fano resonance peak at a normalized frequency value of 0.142 with the transmitted intensity of 76%. Meanwhile, the Fano resonance peak of CO_2_ gas appeared at normalized frequencies of 0.123. has transmitted intensity about 1%, followed by the N_2_O gas it has Fano resonance peak appeared at a normalized frequency of 0.122 with transmitted intensity value of 81% as shown in Fig. 2b. The F(5) quasi-periodic PhC recorded the highest normalized frequency for the CH_4_ followed by CO_2_, and N_2_O with values of 0.273, 0.252, and 0.244 respectively with a transmitted intensity of 32, 80, and 56% for CH_4_, CO_2_, and N_2_O gases as seen in Fig. 2c. On the other hand, the highest normalized frequency range appeared in the case of the FC(7, 1) quasi-periodic structure compared to the periodic and F(5) quasi-periodic PhC gas sensor as observed in Fig. 2f. Thus due to the disorder that occurred in the periodicity in the FC(7, 1) quasi-periodic structure is more than the periodic and F(5) quasi-periodic PhC gas sensor. As a result, a large attenuation occurred for the acoustic sound waves inside the structure as the number of layers increased with disorder arrangement. Furthermore, the CH_4_ has the highest normalized frequency of 0.862 followed by CO_2_, and N_2_O with values of 0.843, and 0.8416, respectively. The appearance of CH_4_ gas at a higher frequency compared to the other gases is due to it having the highest acoustic sound than N_2_O and CO_2_ as shown in Table 2. Secondly, we studied the Fano resonance phenomena that appeared for each gas. It is well known that we live in the resonance world, the Fano resonance is a particularly unusual type of resonance in optics and phononics^32^. It is caused by destructive interference between a discrete quantum state and a continuum band of states at the interface of the Pt, PtS_2_, and gas layer^37,59,60^. Moreover, there was a big difference between our Fano resonance peaks of CO_2_, N_2_O, and CH_4_ gases as shown in Fig. 2 and the normal resonance peaks appeared in different PhC sensors including^61–64^. The Fano resonance peaks are unique with a very sharp line shape and indicating that they have a major impact on sensitivity measures^32^. Our Fano resonance peaks are very symmetric and sharp according to several studies that introduced the Fano resonance transmitted peaks through multilayers phononic crystals including; Ilyasse et al. studied the Fano resonance induced in a 1D solid–fluid phononic crystal^65^. From their results, we show the shape of Fano resonance peaks that appeared. When we compared our Fano resonance peaks with the Ilyasse et al. work we can show that our Fano resonance peaks have strongly asymmetric Fano line shapes as shown in Fig. 2. Also, Xiangli et al. introduced Fano resonance based on surface phonon resonance^66^. According to their results, we can also see that our Fano resonance peaks are more sharp and symmetric than Xiangli et al. work. Further, Oudich et al. proposed a phononic crystal and demonstrated the transmission of the acoustic waves through the structure^67^. Their results introduced the transmitted Fano resonance peaks with very low symmetry and sharpness compared to our Fano resonance peaks. Furthermore, Ting Zhang et al. studied the appearance of Fano resonance mode through 2D sonic crystal^68^. They calculated the transmission versus frequency and the Fano resonance peaks induced inside the band gap. Our results showed very sharp and strong asymmetric Fano line shapes as seen in Fig. 2 compared to Ting Zhang et al. work. The inclusion of Fano resonance in any sensor design often improves the sensitivity and quality factor values, which is the primary reason for including Fano peaks in our sensor design. The Fano-resonance’s asymmetric line form can have a direct effect on the observed high sensitivity value toward certain gases^69^. As introduced in Fig. 2a–f we have significant asymmetric and sharp Fano resonance peaks that indicated the detection of N_2_O, CH_4_, and CO_2_ greenhouse gases by our periodic and quasi-periodic PhC structures. Further, we can show that the abrupt Fano resonance peaks were a major contributor to the suggested gas sensor structure’s high sensitivity records in the GHz frequency band. The Fano resonance peaks changed toward higher frequencies as the acoustic speed of sound of CH_4_, CO_2_, and N_2_O gases increased, as seen in Fig. 2b, c and f. Furthermore, several studies introduced the negative Fano resonance transmission spectra including Cheng Yang et al. who studied the negative transmission of Fano resonance spectra of acoustic waves through an empty hole with an acoustic seal in a wall of finite thickness. They demonstrated that the negative transmission value is due to the acoustic energy is extracted to the hole from a region much larger than the physical dimension of the hole itself. Moreover, they introduced that the negative transmission of Fano resonance peaks indicates that the transmission coefficient is larger than unity. The transmission coefficient is defined as the incident power transmitted fraction within the hole. A coefficient larger than unity showed that the acoustic energy transmitted through the hole exceeds that incident upon hole^59^. In addition, Xin Zhang et al. introduced the transmission of sound within a finite thickness opening with and without an acoustic seal are investigated. Their results showed negative Fano resonance transmitted peaks which indicate that the acoustic energy is flowing into the opening from a region much larger than the opening’s physical dimension. The negative transmission value is because the nominal incident power used in the transmission coefficient definition does not account for the amount of energy injected into the opening^70^. Further, Hongbo et al. studied the negative transmission spectra of the elastic waves through a phononic crystal (PhC) consisting of elliptical steel cylinders embedded in an epoxy matrix^71^. Furthermore, in 2020, we published a paper in the journal of “Nature Scientific Reports” and we explained the negative Fano resonance transmitted peaks that were introduced through a defected phononic crystal gas sensor^16^*.* In this work, our results demonstrated negative Fano resonance transmission spectra. When the acoustic wave interacts with our PhC gas sensor, we considered the gas defect layer as an empty hole and the appearance of negative transmission spectra are due to the acoustic energy is extracted to the gas defect layer from a region (lead and epoxy layers) much larger than the physical dimension of the gas defect layer itself. Also, the negative transmission value introduced a transmission coefficient higher than unity; this represented that the acoustic energy transmitted through the gas defect layer exceeds that incident upon itself^59^. Meanwhile, the negative Fano resonance transmitted modes introduced by periodic and quasi-periodic PhC structures greenhouse gas sensors for the sensing of CO_2_, N_2_O, and CH_4_ gases. In the middle of our sensors structures, there is a cavity that will be filled with sensing gases separately. The gas cavity is treated as an empty hole when the acoustic wave interacts with our optimized structures. So, the appearance of the negative transmitted Fano resonance modes is due to the acoustic energy is extracted to the gas cavity from a region (the Pt and PtS_2_ layers) that is much higher than the physical dimension of the gas cavity itself^59^. Moreover, the negative transmission value revealed a transmission coefficient greater than unity, thus implying that the acoustic energy conveyed through the gas cavity is greater than the energy incident upon the gas cavity^59^. Further, our proposed PhC gas sensors can be introduced theoretically and experimentally easily as using periodic PhC structures for detecting applications were represented in several works of literature^23,72–76^. The sensitivity of the periodic and quasi-periodic phononic crystal gas sensor structures towards N_2_O, CH_4_, and CO_2_ at room temperature as a function of resonance Frequency is calculated below.

### Sensor parameters

Many parameters introduced the performance and efficiency of any sensor, including sensitivity (S), quality factor (Q), and Figure of merit (FOM). These parameters will be obtained by using the following equations^77–79^:

17 $$ S = \Delta f_{res} /\Delta x $$

18 $$ Q = f_{res} /FWHM $$

19 $$ FOM = S/FWHM $$

where resonance frequency represents by $$ f_{res}$$,$$ \Delta f_{res} = f_{{r\left( {gas} \right)}} - f_{{r\left( {air} \right)}}$$, ∆x change of input parameter (density or temperature), and FWHM is the full width at half maximum of the Fano resonance peak.

**Figure 3:**
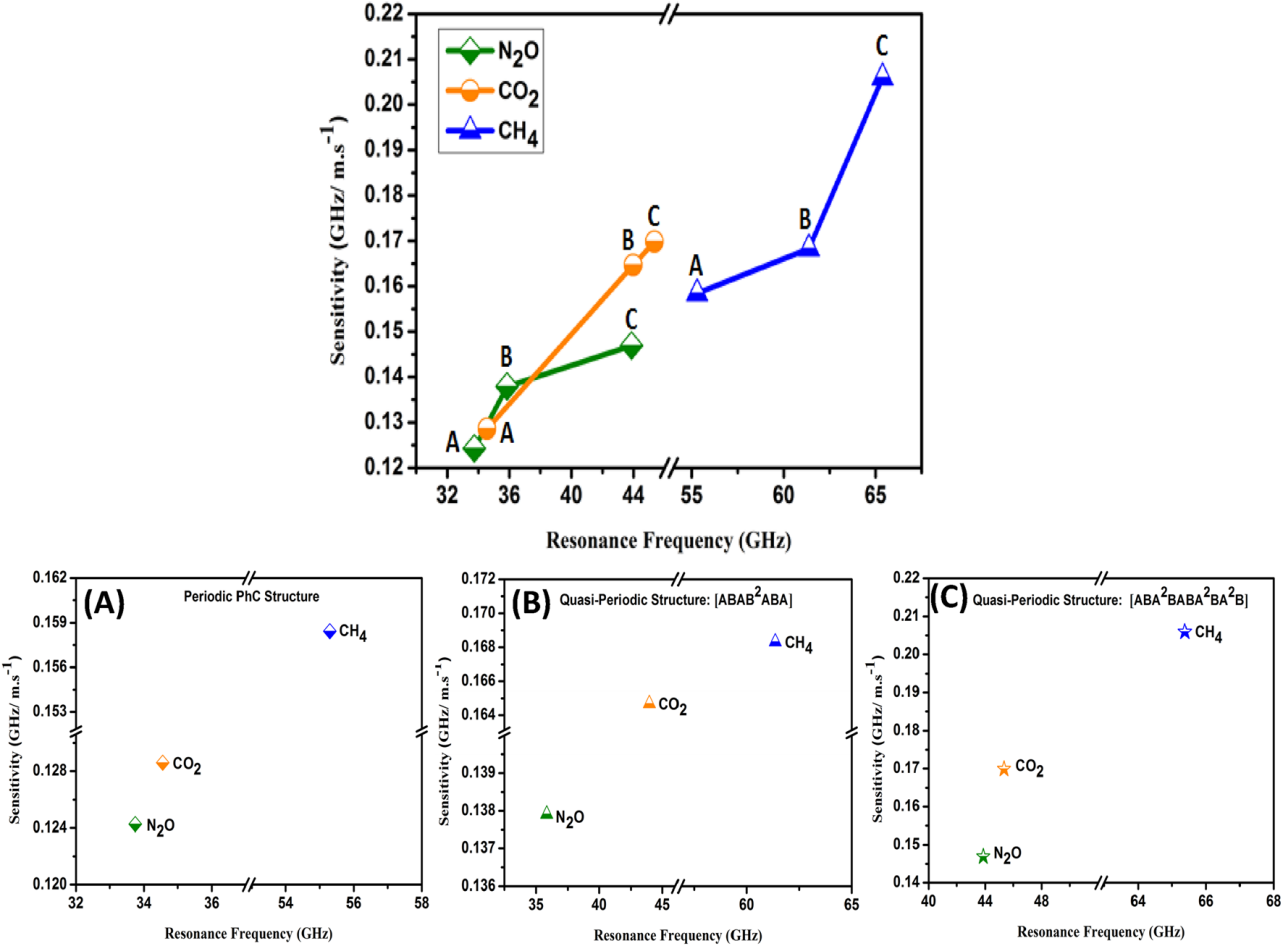
Shows the sensitivity of PhC gas sensor structures towards N_2_O, CH_4_, and CO_2_ greenhouse gases at room temperature as a function of the resonance frequency. (**A**) Periodic PhC structure, (**B**) F(5) quasi-periodic PhC structure, and (**C**) FC(7, 1) quasi-periodic structure.

The sensitivity of periodic and quasi-periodic PhC gas sensor structures towards N_2_O, CH_4_, and CO_2_ greenhouse gases at room temperature is introduced as a function of resonance frequency as seen in Fig. 3. We used Eq. (17) to provide the sensitivity of our PhC gas sensor structures and the results are introduced in Fig. 3. The highest sensitivity represents CH_4_ followed by CO_2_ and N_2_O gases by the periodic and quasi-periodic PhC gas sensor structures. Comparing the three structures the FC(7, 1) quasi-periodic PhC gas sensor structures recorded the highest sensitivity values of 2.059, 1.698, and 1.469 (GHz/m.s^−1^) towards CH_4_, CO_2,_ and N_2_O gases respectively as observed in Fig. 3C. Thus, it introduced higher resonance frequency values for the three gases than the periodic and F(5) quasi-periodic PhC gas sensor structures. The highest frequency values appeared when we examined the FC(7, 1) quasi-periodic structure due to the disorder that occurred in the periodicity being more than the periodic and F(5) quasi-periodic PhC structures. As a result, a large attenuation occurred for the acoustic sound waves inside the structure as the number of layers increased with disorder arrangement. On the other side, the periodic PhC structure sensor introduced the lowest sensitivity towards CH_4_, CO_2,_ and N_2_O gases. In addition, we have seen that, from Eq. (17), the greenhouse gases sensitivity depends on the resonance frequency, as the fact of with raising the frequency, the sensitivity will be also increased. Our designed PhC gas sensors structures based on Fano resonance represents higher sensitivity towards CH_4_ gas than CO_2_ and N_2_O greenhouse gases.

### The quality factor of the periodic and quasi-periodic PhC gas sensor structures towards N_2_O, CH_4_, and CO_2_ gases

**Figure 4:**
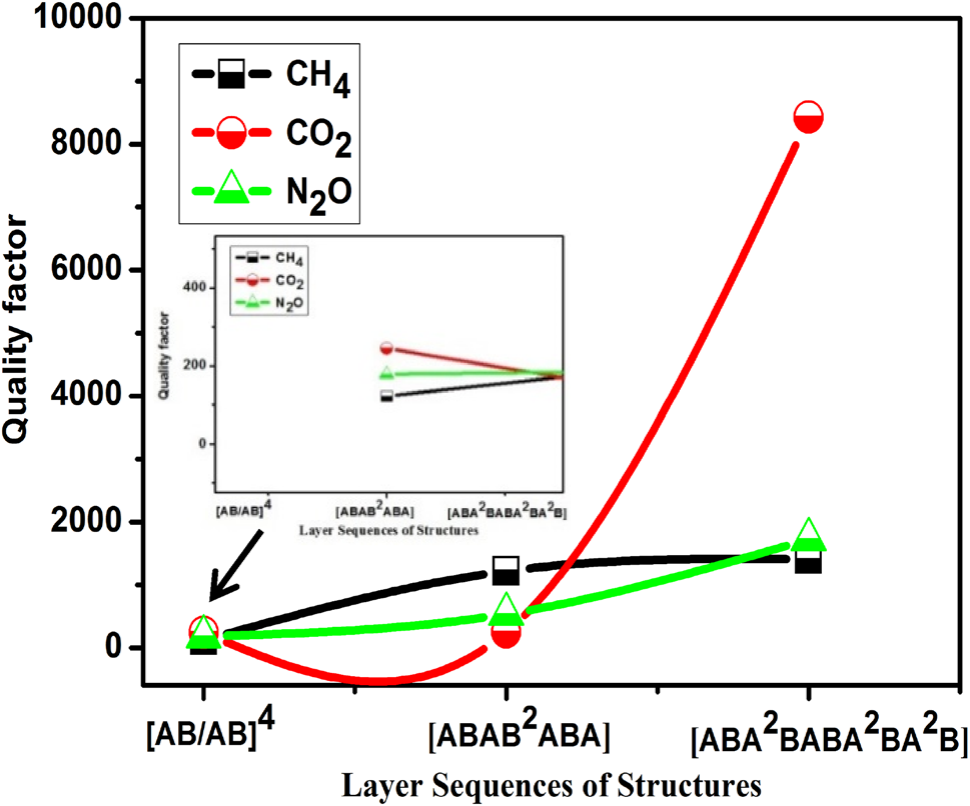
Shows the Quality factor of the periodic and F(5), FC(7, 1) quasi-periodic PhC gas sensor structures towards N_2_O, CH_4_, and CO_2_ gases.

Sharp Fano resonance peaks with a high-quality factor should be appeared to introduce a high-efficiency gas sensor. The high-quality factor values denote a precise sensor measurement. The S, FOM, and Q sensing parameters values are provided by using Eqs. (17)–(19) at the Fano resonance peak of each gas. The quality factor of periodic and quasi-periodic PhC gas sensor structures has been illustrated in Fig. 4. As shown in the Figure, the highest quality factor introduced by the FC(7, 1) followed by F(5) quasi-periodic PhC gas sensor structure. Meanwhile, the periodic structure with layer sequences of [AB/AB]^4^ recorded the lowest quality factor for N_2_O, CH_4_, and CO_2_ greenhouse gases. The FC(7, 1) quasi-periodic gas sensor recorded the highest Q value of 8430 for CO_2_ gas followed by N_2_O, CH_4_ of about 1724, and 1403 respectively. The CO_2_ gas has the highest Q value because it has the lowest FWHM among the other gases, but the CH_4_ gas has the lowest Q because its FWHM is greater. As a result, the FWHM values of gases are as follows ${\text{FWHM}}_{{{\text{CH}}_{4} }} > {\text{FWHM}}_{{{\text{N}}_{2} {\text{O}}}} > {\text{FWHM}}_{{{\text{CO}}_{2} }}$. The detected greenhouse gases’ high Q values are due to a small broadening in their Fano resonance peaks. On the other side, the F(5) quasi-periodic PhC gas sensor introduced the highest Q value of 1219 for CH_4_ gas followed by N_2_O, and CO_2_ of about 536, and 251.5 respectively. The Q values decreased in the case of the F(5) gas sensor due to the Fano resonance peaks related to CH_4_, N_2_O, and CO_2_ gases having large FWHM values of 0.21, 0.52, and 1.05 GHz respectively. Further, the lowest Q values for CO_2_, N_2_O, and CH_4_ gases were recorded by the periodic structure of [AB/AB]^4^ PhC gas sensor with values of 245, 178.1, and 122 respectively as seen in Fig. 4. Thus, due to the highest FWHM of the Fano resonance peaks were introduced for CH_4_, N_2_O, and CO_2_ gases with values of 1.1, 0.84, and 0.5 GHz. The detected greenhouse gases’ low Q values are due to a large broadening in their Fano resonance peaks.

### The effect of FWHM on the FOM and Q of the periodic and quasi-periodic PhC gas sensor structures towards N_2_O, CH_4_, and CO_2_ gases

**Figure 5:**
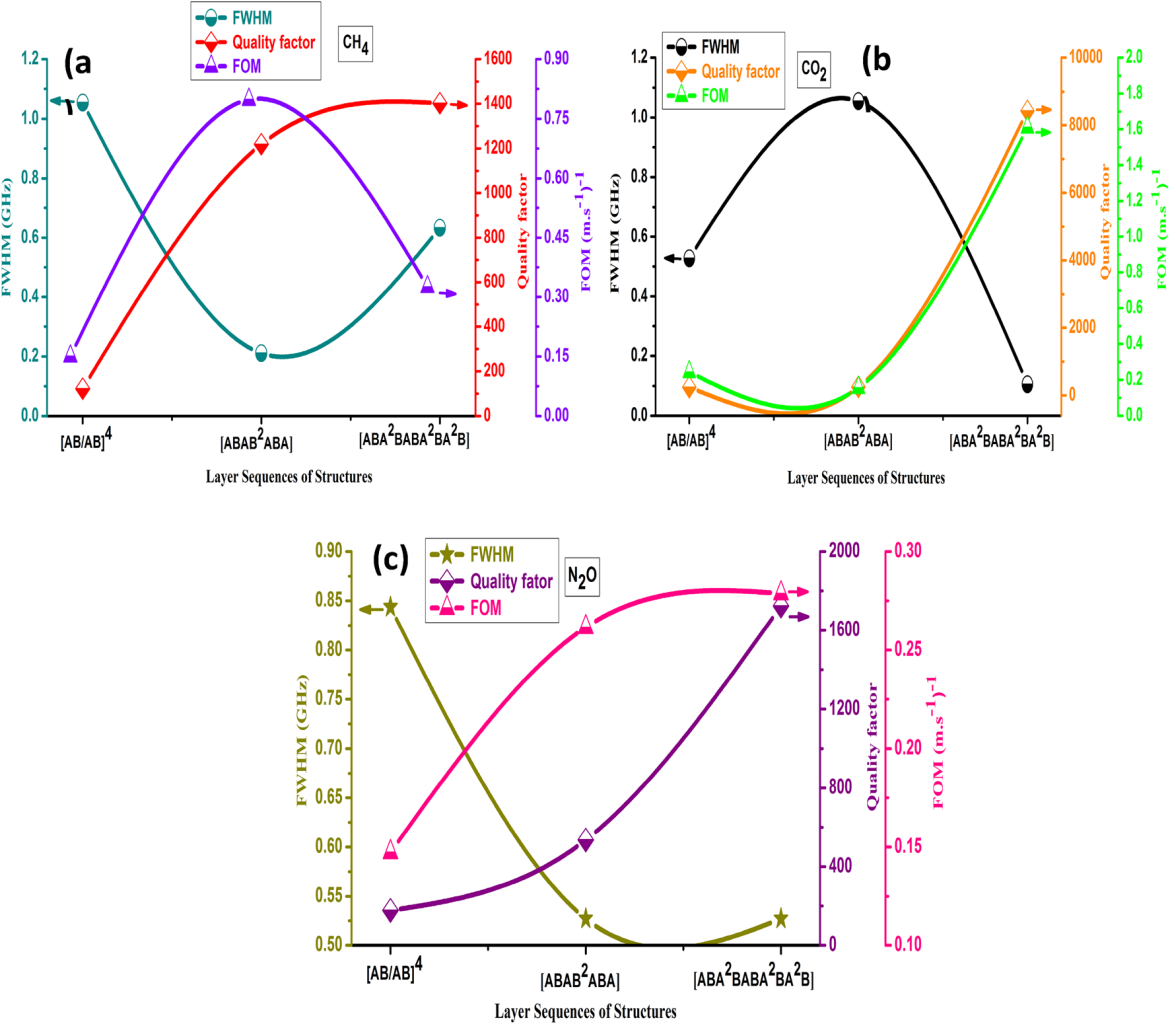
Shows the Quality factor of the periodic and F(5), FC (7, 1) quasi-periodic gas sensor structures towards (**a**) CH_4_, (**b**) CO_2_, and (**c**) N_2_O greenhouse gases.

We studied the effect of FWHM of the Fano resonance peaks of the N_2_O, CH_4_, and CO_2_ gases on the Q, FOM of the periodic and F(5), FC(7, 1) quasi-periodic PhC gas sensor structures. It is well known that the PhC sensor detection accuracy is inversely proportional to the FWHM of the Fano resonance transmitted peak^80^. As observed in Fig. 5a, b, c, the Q and FOM have been affected by the FWHM of the Fano resonance peak of each gas tested by the periodic and quasi-periodic structures. The highest Q was recorded for CH_4_ gas by FC(7, 1) quasi-periodic structure, as according to Eq. (9) and Fig. 2C the highest Fano resonance frequency was recorded for CH_4_ gas. On the other side, the lowest Q value is recorded by the periodic PhC gas sensor as shown in the Figure. Moreover, the FOM of CH_4_ has the highest value of 0.78 (m.s^−1^)^−1^ for the F(5) followed by FC(7, 1) quasi-periodic PhC gas sensor as observed in Fig. 5a. On the other hand, as seen in Fig. 5a the lowest Q and FOM values of 122 and 0.15 (m.s^−1^)^−1^ were recorded by periodic PhC gas sensor, due to the highest FWHM value of 1.05 GHz, and the Q and FOM inversely proportional to the FWHM of the Fano resonance transmitted peak according to Eqs. (9, 10)^80^. For the CO_2_ gas, the FC(7, 1) quasi-periodic PhC structure introduced the highest Q and FOM values of 8430 and 1.61 (m.s^−1^)^−1^ respectively, due to the lowest FWHM value of 0.1 GHz as represented in Fig. 5b. Similarly, the FC(7, 1) quasi-periodic PhC structure recorded the highest Q and FOM values of 1724 and 0.28 (m.s^−1^)^−1^ respectively for the N_2_Ogas, due to the lowest FWHM value of 0.5 GHz as introduced in Fig. 5c. From our results as represented in Fig. 5a, b, c we can see that the highest FOM was observed for the CH_4_ gas compared to other gases.

### Effect of temperature on fano resonance peaks position of the FC(7, 1) quasi-periodic PhC gas sensor towards CH_4_ gas

**Figure 6:**
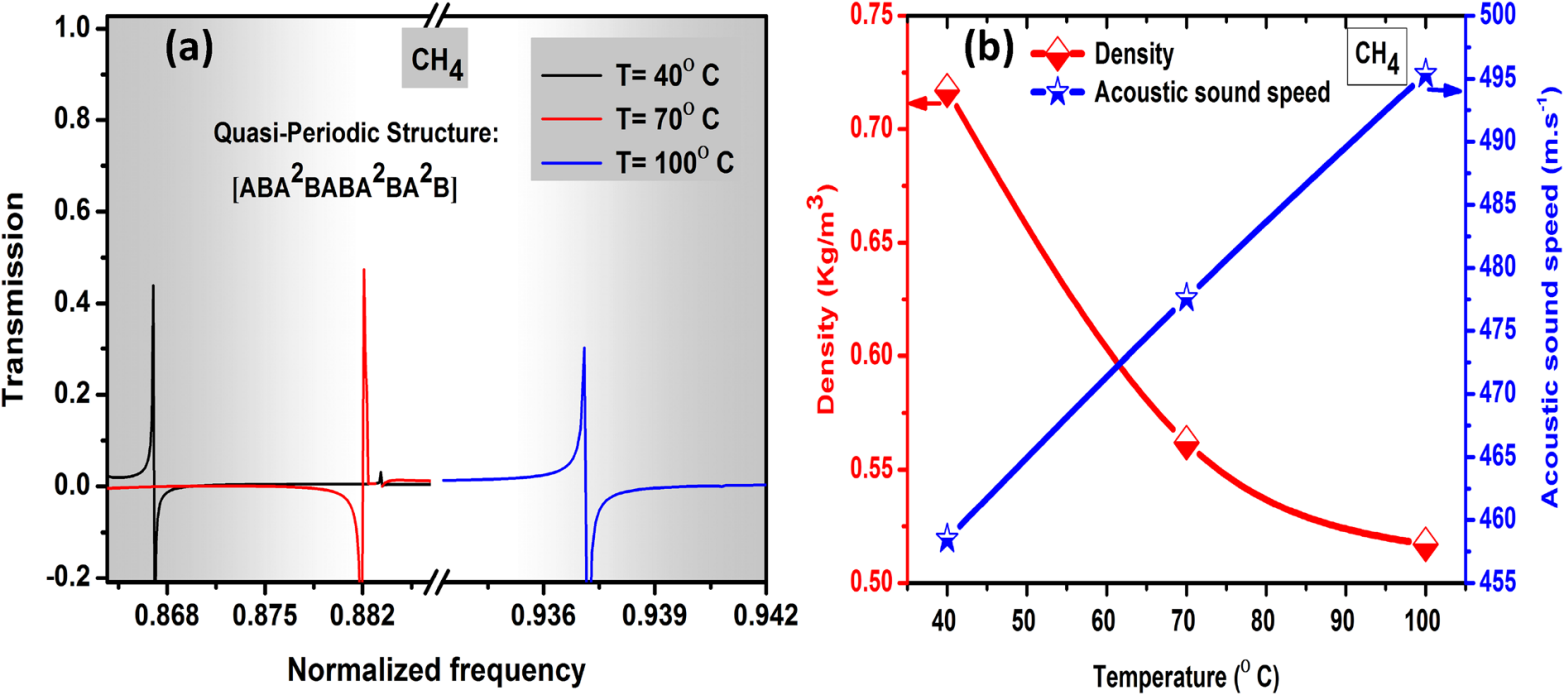
Illustrates the temperature effect on the (**a**) Fano resonance peak position of FC(7, 1) quasi-periodic gas sensor towards CH_4_ gas (**b**) change of CH_4_ gas acoustic properties with different temperatures.

As our results introduced the FC(7, 1) quasi-periodic structure is the best gas sensor structure represented high sensitivity and Fano resonance frequency for the N_2_O, CH_4_, and CO_2_ gases. Moreover, the highest sensitivity, Fano resonance frequency, and FOM were recorded by FC(7, 1) quasi-periodic structure for the CH_4_ gas. In this part, we studied the effects of temperature on the Fano resonance peaks position of CH_4_ gas through the FC(7, 1) quasi-periodic gas sensor, the propagation of the acoustic wave through the CH_4_ gas cavity at 40, 70, and 100 °C as observed in Fig. 6a. In addition, we introduced the temperature effect on the CH_4_ gas acoustic properties which also affect the Fano resonance peaks. Temperature is known to have a direct effect on the density and acoustic sound speed of gases, with increasing the temperature the acoustic sound speed of gases increased as well as the gases density decreased as seen in Fig. 6b and Table 3^81,82^. As a result, the position of CH_4_ gas’s Fano resonance peak shifted to the high-frequency range with increasing the temperature as shown in Fig. 6a^83^.

**Table 3:** The acoustic properties of CH_4_ gas and Fano resonance frequency values at 40, 70, and 100 °C.

Gas	Temperature (°C)	Density (kg/m^3^)	Acoustic speed (m/s)	Resonance frequency (GHz)
CH_4_	40	0.717	458.4	70.7
	70	0.562	477.6	930.2
	100	0.517	495.4	988.17

### The effect of temperature on the sensitivity and fano resonance frequency of the FC(7, 1) quasi-periodic PhC gas sensor towards CH_4_ gas

**Figure 7:**
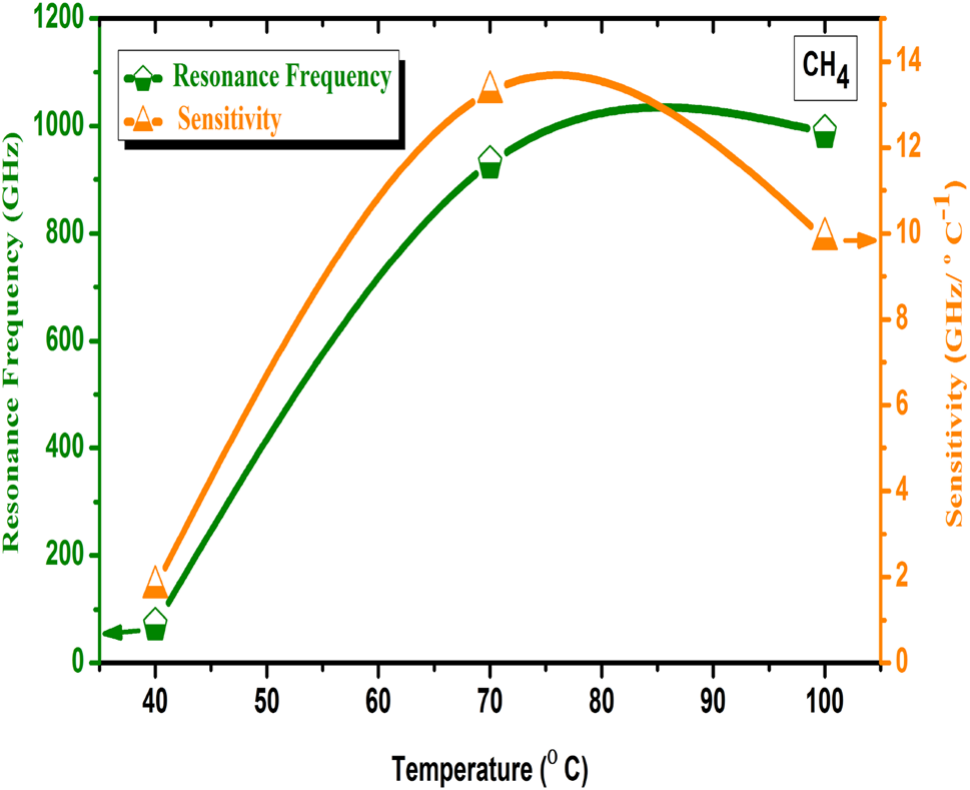
Effects of temperature on the resonance frequency and sensitivity of the FC(7, 1) quasi-periodic gas sensor towards CH_4_ gas at 40, 70, and 100 °C.

The temperature has a significant impact on the FC(7, 1) quasi-periodic gas sensor’s performance. It has an impact on detection accuracy, which is introduced as the sensor’s ability to provide the sensing medium’s resonance frequency. The sensitivity appears to decrease with increasing the temperature, as shown in Fig. 7. According to Eq. (17), the gas sensor sensitivity is directly proportional to ∆$$f_{res} $$^82^, as a result of increasing ∆$$f_{res}$$ the sensitivity increased as well. Actually, the temperature is known to have a direct effect on the density and acoustic sound speed of gases, with increasing the temperature the acoustic sound speed of gases increased and the gases density decreased as seen in the Fig. 6b and Table 3^81,82^. As a result, the position of CH_4_ gas’s Fano resonance peak shifted to the high-frequency range with increasing the temperature as shown in Fig. 6a^83^. Also, as shown in Fig. 7 it’s showed that at 70 °C the FC(7, 1) quasi-periodic gas sensor recorded the highest sensitivity for CH_4_ gases with the value of 13.3 (GHz/°C) while the lowest sensitivity was introduced at 40 °C with a value of 1.77 (GHz/°C). As the ∆$$f_{res}$$ at 70 °C has a value higher than 40 °C as given in Fig. 7 and Table 3.

### Effect of temperature on the quality factor, FOM of the FC(7, 1) quasi-periodic PhC gas sensor towards CH_4_ gas

**Figure 8:**
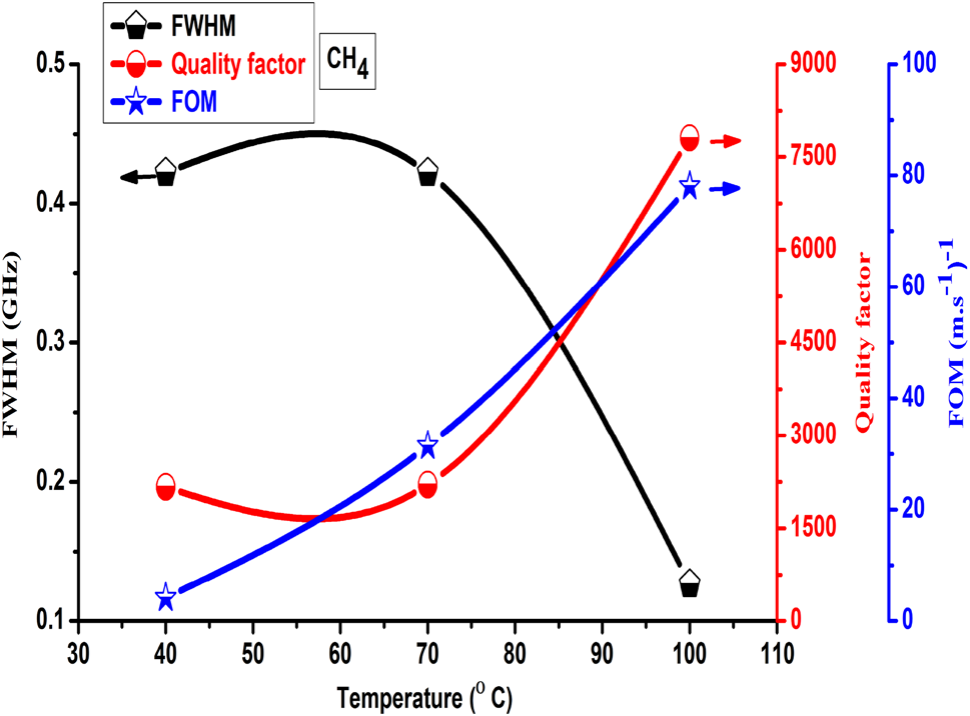
Effects of temperature on the quality factor and FOM of the FC(7, 1) quasi-periodic gas sensor towards CH_4_ gas at 40, 70, and 100 °C.

Figure 8 demonstrated the temperature effects on the quality factor and FOM of the FC(7, 1) quasi-periodic gas sensor towards CH_4_ gas at 40, 70, and 100 °C. The quality factor introduced the Fano resonance peaks sharpness, the higher the quality factor, the sharper the peak^84^. As observed in Fig. 8, the highest quality factor and FOM recorded towards CH_4_ have values of 7809 and 78.1 (m.s^−1^)^−1^ respectively at 100 °C, followed by 70 °C with Q and FOM values of 2205 and 31.5 (m.s^−1^)^−1^ respectively. Thus, the lowest FWHM introduced for CH_4_ gas at 100 °C with a value of 0.13 GHz followed by 70 °C about 0.42 GHz as represented in Fig. 8. On the other side, as demonstrated in Fig. 8 the lowest Q and FOM appeared at 40 °C with values of 2167 and 4.1 (m.s^−1^)^−1^ respectively. Thus, due to the lowest values of the sensitivity and resonance frequency recorded by the FC(7, 1) quasi-periodic gas sensor towards CH_4_ gas at 40 °C, which in turn lead to a decrease in the Q and FOM as well based on Eqs. (18, 19). From our results, we can see that the FC(7, 1) quasi-periodic will be introduced a novel Q and FOM gas sensor towards CH_4_ gas at 100 and 70 °C.

## Conclusion

In summary, the performance of the periodic and quasi-periodic PhCs structures has been demonstrated for greenhouse gas sensing applications. Fano resonance modes have been observed in Pt/PtS_2_ utilized ultra-sensitive gas sensors towards CO_2_, N_2_O, and CH_4_ gases. Our study approved that the acoustic properties mismatch and the sequences of layers play a significant role in the control of Fano resonance and transmission passbands. The generalized Fibonacci (FC(7, 1)) quasi-periodic structure introduced the best gas sensor structure and represented the highest sensitivity for CO_2_, N_2_O, and CH_4_ gases compared to periodic and simple Fibonacci (F(5)) phononic crystal structures. Moreover, for the first time very shark Fano resonance modes were observed in the investigated gas sensor structures, resulting in high Fano resonance frequency, novel sensitivity, quality factor, and figure of merit values for all greenhouse gases. The highest sensitivity was introduced by FC(7, 1) quasiperiodic structure for the CH_4_ with a value of 2.059 (GHz/m.s^−1^). Further, the temperature effect on the position of Fano resonance modes introduced by FC(7, 1) quasi-periodic PhC gas sensor towards CH_4_ gas has been introduced in detail. The results show the highest sensitivity at 70 °C with a value of 13.3 (GHz/°C). Moreover, the highest Q and FOM recorded towards CH_4_ have values of 7809 and 78.1 (m.s^−1^)^−1^ respectively at 100 °C.
